# Screen-based behaviour in school-aged children with long-term illness

**DOI:** 10.1186/s12889-016-2804-8

**Published:** 2016-02-09

**Authors:** Daniela Husarova, Andrea Madarasova Geckova, Lukas Blinka, Anna Sevcikova, Jitse P. van Dijk, Sijmen A. Reijneveld

**Affiliations:** 1 Department of Health Psychology, Faculty of Medicine, P.J. Safarik University in Kosice, Tr. SNP 1, Kosice, 040 01 Slovak Republic; 2 Graduate School Kosice Institute for Society and Health, Faculty of Medicine, P.J. Safarik University in Kosice, Tr. SNP 1, Kosice, 040 01 Slovak Republic; 3 Center for Kinanthropology Research, Institute of Active Lifestyle, Faculty of Physical Culture, Palacky University in Olomouc, Tr. Miru 115, Olomouc, 77111 Czech Republic; 4 Olomouc University Social Health Institute, Palacky University Olomouc, Tr. Miru 115, Olomouc, 77111 Czech Republic; 5 Institute for Research on Children, Youth and Family, Faculty of Social Studies, Masaryk University Brno, Joštova 10, 602 00 Brno, Czech Republic; 6 Department of Community & Occupational Health, University of Groningen, University Medical Center Groningen, A. Deusinglaan 1, 9713 AV Groningen, The Netherlands

**Keywords:** Long-term illness, Asthma, Learning disabilities, Internet, Watching TV, Playing computer games, Working with a computer, Adolescents

## Abstract

**Background:**

Evidence is lacking on the screen-based behaviour of adolescents with a chronic condition. The aim of our study was to analyse differences in screen-based behaviour of adolescents by long-term illness, asthma and learning disabilities.

**Methods:**

We used data from the cross-sectional Health Behaviour of School-aged Children study collected in 2014 among Slovak adolescents (age 13 to 15 years old, *N* = 2682, 49.7 % boys). We analysed the associations between screen-based behaviour and long-term illness, asthma and learning disabilities using logistic regression models adjusted for gender.

**Results:**

We found no associations between screen-based behaviour and long-term illness, except that children with asthma had a 1.60-times higher odds of excessively playing computer games than healthy children (95 % confidence interval of odds ratio (CI): 1.11–2.30). Children with learning disabilities had 1.71-times higher odds of risky use of the Internet (95 % CI: 1.19–2.45).

**Conclusion:**

Adolescents with a long-term illness or with a chronic condition or a learning disability do not differ from their peers in screen-based activities. Exceptions are children with asthma and children with learning disabilities, who reported more risky screen-based behaviour.

## Background

Electronic devices play a major role in the lives of contemporary children, but this may have negative effects on their physical or psychological health [1–3]. Recent evidence suggests that children are involved in a wide range of online activities, such as doing school work, playing computer games, social networking and messaging [4], and many of them exceed the recommended time spent with these activities [5, 6]. Excessive spending time on the Internet or online gaming, together with other particular personal aspects [7], might precede problematic Internet use with behavioural or social problems [8]. Similarly, all these problems can be deepened by problematic Internet use. Therefore, the motivation for excessive and problematic media may be in a reciprocal relationship, as suggested by Valkenburg and Peter [9] or Slater [10].

According to the WHO [11], the prevalence of chronic conditions generally among adolescents is high. One of the most common chronic conditions with an increasing trend is e.g. asthma [12]. The presence of such a health condition requires management of the condition and patient adherence to daily treatment. This limits many areas of an adolescent’s everyday life, including his or her family, peers or school [13]. Moreover, research indicates that children with different types of chronic conditions are highly involved in a sedentary lifestyle [14] because of limitations in many other activities [15]. However, children might not perceive the impact of the chronic condition on their activities and socialization [16], which could be associated with a good health care system or with psychosocial factors of the individuals themselves [17]. Children use of electronic media, including Internet and video gaming, has increased also among children with health condition like ADHD. The Internet environment and virtual reality offers very attractive features for them. It provides very broad content for potential stimulations or various activities in simultaneously open windows, which might lead to fixation to the online world. Furthermore, video games offer immediate rewards with a strong incentive to increase the reward by trying the next level [18, 19].

Generally speaking, evidence is lacking in regard to screen-based behaviour among children with a chronic condition. In our study we focused on children with long-term illness, asthma and learning disabilities, who are at the greater risks of lower school performance and involvement in sedentary behaviour [14, 20]. Therefore, the aim of the present study was to analyse differences in the screen-based behaviour of adolescents by long-term illness, asthma and learning disabilities.

## Methods

### Sample and procedure

We used data from the Health Behaviour in School-aged Children (HBSC) study conducted in 2014 in Slovakia. To obtain a representative sample, we used two-step sampling. In the first step, 151 larger and smaller elementary schools located in rural as well as in urban areas from all regions of Slovakia were asked to participate. These were randomly selected from a list of all eligible schools in Slovakia obtained from the Slovak Institute of Information and Prognosis for Education. In the end, 130 schools agreed to participate in our survey (response rate: 86.1 %). In the second step, we obtained data from 10,179 adolescents from the 5th to the 9th grades (response rate: 78.8 %). Questionnaires containing measurement on excessive use of interente were randomly distributed in adolescents 13 years and older (7th, 8th and 9th grade) with aim to keep collect data of at least half of them. Therefore,the final sample comprises 2682 adolescents (mean age: 14.11; 49.7 % boys),who filled the questionnaire which contain also measurement on excessive use of internet.

The study was approved by the Ethics Committee of the Medical Faculty at the P. J. Safarik University in Kosice. Procedure of approvement includes assessment of the protocol of the HBSC study which contains information about the passive consent procedure. Parents were informed about the study via the school administration (explanation of study and consent through the children or on parent-teachers meeting) and could opt out if they disagreed with their child’s participation. Participation in the study was fully voluntary and anonymous, with no explicit incentives provided for participation. Questionnaires were administered by trained research assistants in the absence of a teacher during regular class time.

### Measures

*Screen-based activities*, represented by watching TV, playing computer games and working with a computer, were assessed using three separate items. Watching TV was measured by the question: “How many hours a day, in your free time, do you usually spend watching television, videos (including YouTube or similar services), DVDs and other entertainment on a screen?” Computer gaming was measured by asking: “How many hours a day, in your free time, do you usually spend playing games on a computer, gaming console, tablet (like iPad), smartphone or other electronic devices (not including moving or fitness games)? And computer work was assessed by asking: “How many hours a day, in your free time, do you usually spend using electronic devices such as computers, tablets (like iPad) or smartphones for other purposes, for example, homework, e-mailing, tweeting, facebook, chatting, surfing the Internet” [21]. Responses were dichotomized into two categories of children: those who spent less than 2 h per day and those who spent 2 or more hours per day on screen-based activities, as AAP recommended that children should not spend time with media no more than 1 to 2 h per day [22].

Moreover, *excessive Internet use* was measured using five items focused on different types of behaviour as a consequence of spending excessive time on the Internet. Participants indicated how often they experience the following situations in the last 12 months: “I did not eat or sleep because of the Internet.”; “I felt uncomfortable when I could not be on the Internet.”; “I found myself surfing the Internet, even though I did not enjoy it.”; “I neglected my family, friends, school work or hobbies because of the time spent on the Internet.”; “I tried to reduce the time spent on the Internet, but without success.” Responses were measured on a 4-point scale: very often, often, rarely, never [23]. Those who reported to experience the particular situation very of often or often during past year were considered to “have a symptom”. Then we divided adolescents on those who do not have any symptom excessive use of internet and those who have at least one symptom of excessive use of internet.

*Long-term illness* prevalence was assessed using the item: “Do you have a long-term illness, disability or medical condition (like diabetes, arthritis, allergy or cerebral palsy) that has been diagnosed by a doctor?” with “yes” and “no” as the response categories [24]. The response used in statistical analyses referred to the occurrence of long-term illness.” Besides this question we asked adolescents if they have asthma and learning disabilities (dyslexia, dysgraphia, orthography, dyscalculia) confirmed by a doctor.

### Statistical analysis

First, we described the sample using descriptive statistics. Next, the relationships between screen-based behaviour and long-term illness, asthma and learning disabilities were explored separately using logistic regression models adjusted for gender. Interactions of the effects of gender and health condition (e.g. long-term illness, asthma, learning disability respectively) on screen-based behaviour were assessed, but none of them were found to be significant (not presented). All analyses were performed using SPSS version 21.0.

## Results

Around 20 % of adolescents had a long-term illness or medical condition that has been diagnosed by a doctor (Table 1). Moreover, more than half of adolescents exceeded the recommended time for screen-based activities, such as watching TV, playing PC games and computer work. The prevalence of screen-based activities and excessive use of the Internet was relatively similar for children with and without a chronic condition or learning disability (Table 2). Children with a long-term illness and learning disability did not differ from their peers in screen-based activities, such as watching TV, playing computer games and working with a computer. However, children with asthma had 1.59-times higher odds of excessive playing of computer games in comparison with their peers (Table 2). Children reporting learning disabilities, but not reporting long-term illness or asthma, had 1.71-times higher odd of excessive use of internet. Interactions of the effects of gender and long-term illness, asthma or learning disabilities were not statistically significant (not shown).

**Table 1:** Prevalence of screen-based behavior and long-term illness among school-aged children

		*N* (%)
Watching TV	≥2 h	1,723 (71.1)
Playing PC games	≥2 h	1,198 (49.3)
Computer work	≥2 h	1,483 (61.1)
Excessive use of internet	At least one symptom	810 (35.2)
Long-term illness	yes	574 (21.6)
Asthma	yes	158 (6.0)
Learning disability	yes	174 (6.6)

**Table 2:** Prevalence and odds ratios (95 % CI) for excessive screen-based behaviour among adolescents with and without long-term illness, asthma and learning disabilities

		Watching TV (≥2 h)		Playing PC games (≥2 h)		Computer work (≥2 h)		Excessive use of internet (≥1 symptom)	
		*N* (%)	OR (95 % CI)	*N* (%)	OR (95 % CI)	*N* (%)	OR (95 % CI)	*N* (%)	OR (95 % CI)
Long-term illness	Yes	369 (72.4)	1.08 (0.87–1.35)	256 (50.5)	1.12 (0.91–1.38)	306 (60.4)	0.96 (0.78–1.17)	190 (38.9)	1.23 (0.99–1.51)
	No	1,346 (70.8)	1 (ref)	936 (49.0)	1 (ref)	1,168 (61.3)	1 (ref)	615 (34.1)	1 (ref)
Asthma	Yes	104 (74.8)	1.23 (0.83–1.82)	81 (57.4)	*1.59 (1.11–2.30)	92 (65.7)	1.23 (0.86–1.77)	48 (35.8)	1.02 (0.71–1.47)
	No	1,600 (70.9)	1 (ref)	1,101 (48.7)	1 (ref)	1,370 (60.7)	1 (ref)	86 (64.2)	1 (ref)
Learning disability	Yes	90 (67.2)	0.82 (0.56–1.19)	71 (52.6)	0.95 (0.66–1.37)	85 (63.4)	1.13 (0.79–1.63)	60 (46.9)	**1.71 (1.19–2.45)
	No	1,607 (71.2)	1 (ref)	1,110 (49.1)	1 (ref)	1,373 (60.9)	1 (ref)	740 (34.6)	1 (ref)

*p < 0.05 **p < 0.01

*N* = number of children with and without long-term illness, asthma and learning disabilities in each screen-based behaviour and excessive use of internet

## Discussion

Our objective was to explore the association between screen-based behaviour and the occurrence of long-term illness, asthma and learning disabilities among school-aged children. We found that adolescents with asthma were more likely to play computer games than their peers without any chronic conditions. The study also showed adolescents with a learning disability were at greater risk of excessive Internet use.

Our findings corroborate prior research that children with chronic conditions incline toward sedentary behaviour [14] and expand current knowledge by identifying which screen-based activities stand for their preferred sedentary behaviours. The association between asthma and playing computer games poses a new question of whether involvement in computer games represents an alternative leisure activity that parents offer to their children in order to have them under greater surveillance. However, the explanation may also lie in the motivation of the children. Some studies suggest a relationship between asthma and increased sedentary behaviour e.g. leading to obesity [25–27]. Due to a lack of physical activities, asthmatic children may have lower self-esteem and self-efficacy and greater mood difficulties, which has been partially shown in the literature [28, 29]. Computer gaming is often classified as a mood-management activity which increases one’s own feelings of competence [30–32] and which may be popular among asthmatic children due to the substitution and coping strategy.

More than half of school-aged children exceed recommended time spent on screen-based activities, and adolescents with chronic conditions were rather similar to their peers. Other studies on children have also shown an increased amount of time devoted to screen-based activities [33, 34]. This pattern of spending their leisure-time thus seems to be a general trend characteristic for this young generation. It may be a result of the development of new technologies surrounding adolescents in everyday life, including school or family, which may increase the risk of sedentary behaviour.

In addition, the present study showed that adolescents with a learning disability are at higher risk of developing symptoms of excessive Internet use in comparison with their peers. There might be two alternative explanations. According to the first one, learning disability and excessive Internet use may have a common denominator that is impaired executive functions [35, 36]. It is also possible that excessive Internet use is an outcome of a maladaptive coping strategy in the sense that these children might be compensating for their shortcomings by being active online. There is a growing body of literature reporting the relationship between ADHD and excessive Internet use [19, 37]. Although learning disabilities and ADHD are not identical, they are closely related and overlapping – e.g. children of both groups have attentional difficulties and are easily bored [38]. Moreover, Cook et al. [39] indicate that multiple factors, like poor motor skills and executive function deficits in children with learning disabilities, might contribute to low levels of physical activity and to high levels of sedentary behaviour subsequently. This offers a possible explanation as to why children with learning disabilities may become fixated to the online world and why these children should be a prime target of prevention. Although further research with more sophisticated design is needed, it is worth mentioning that the present study opens a gate to this issue.

The most important strengths of the study are the representativeness of our sample of adolescents and the use of internationally recognized instruments. In addition, our study provides important information with regards to screen-based behaviour in children with long-term illness, which is lacking in the literature. Nevertheless, some limitations need to be considered. Firstly, we used only self-reported data. Measurement of long-term illness is very general and might comprise a very heterogeneous group of health problems with regard to type as well as severity, and consequently also with regard to their impact on daily activities. Secondly, our study has a cross-sectional design; therefore, we are unable to formulate conclusive statements about causality. Finally, our sample did not include children with long-term illness who are not able to attend school regularly because of their health condition.

## Conclusion

The findings show that adolescents with long-term illness or chronic condition do not differ from their peers in screen-based behaviour, with exception of asthmatic children playing computer games more often and children with learning disabilities being more prone to excessive Internet use. However, further research focused on separate clinical groups but using a measurement used in representative samples might bring more insight and understanding regarding the lifestyle of children who are excluded from mainstream schools. Based on our results, it seems to be important to assess determinants of their social environment, which could be helpful in developing interventions to reduce involvement in excessive screen-based behaviour and the subsequent negative consequences.
